# BDS Precise Point Positioning for Seismic Displacements Monitoring: Benefit from the High-Rate Satellite Clock Corrections

**DOI:** 10.3390/s16122192

**Published:** 2016-12-20

**Authors:** Tao Geng, Xing Su, Rongxin Fang, Xin Xie, Qile Zhao, Jingnan Liu

**Affiliations:** GNSS Research Center, Wuhan University, 129 Luoyu Road, Wuhan 430079, China; gt_gengtao@whu.edu.cn (T.G.); longtian00@163.com (X.S.); xiexin@whu.edu.cn (X.X.); zhaoql@whu.edu.cn (Q.Z.); jnliu@whu.edu.cn (J.L.)

**Keywords:** BDS, precise point positioning, seismic displacements, Nepal earthquake, satellite clock corrections

## Abstract

In order to satisfy the requirement of high-rate high-precision applications, 1 Hz BeiDou Navigation Satellite System (BDS) satellite clock corrections are generated based on precise orbit products, and the quality of the generated clock products is assessed by comparing with those from the other analysis centers. The comparisons show that the root mean square (RMS) of clock errors of geostationary Earth orbits (GEO) is about 0.63 ns, whereas those of inclined geosynchronous orbits (IGSO) and medium Earth orbits (MEO) are about 0.2–0.3 ns and 0.1 ns, respectively. Then, the 1 Hz clock products are used for BDS precise point positioning (PPP) to retrieve seismic displacements of the 2015 Mw 7.8 Gorkha, Nepal, earthquake. The derived seismic displacements from BDS PPP are consistent with those from the Global Positioning System (GPS) PPP, with RMS of 0.29, 0.38, and 1.08 cm in east, north, and vertical components, respectively. In addition, the BDS PPP solutions with different clock intervals of 1 s, 5 s, 30 s, and 300 s are processed and compared with each other. The results demonstrate that PPP with 300 s clock intervals is the worst and that with 1 s clock interval is the best. For the scenario of 5 s clock intervals, the precision of PPP solutions is almost the same to 1 s results. Considering the time consumption of clock estimates, we suggest that 5 s clock interval is competent for high-rate BDS solutions.

## 1. Introduction

Precise point positioning (PPP) is a global navigation satellite system (GNSS) positioning method to calculate precise positions at centimeter- or even millimeter-level accuracy with a single GNSS receiver using precise satellite orbit and clock products [1,2]. Over the past decades, PPP using Global Positioning System (GPS) has been proved as an effective approach for the measurement of arbitrarily large ground motions and seismic displacements induced by earthquakes [3,4,5,6,7].

BeiDou Navigation Satellite System (BDS) began to offer positioning, navigation, and timing services in the Asia-Pacific region at the end of 2012. It is aimed at serving global users upon its completion by 2020. Many studies have been conducted to investigate the performance of BDS precise positioning. With three geostationary Earth orbit (GEO) and three inclined geosynchronous orbit (IGSO) satellites, Shi et al. [8] demonstrated that precise relative positioning at an accuracy of 1–2 cm in horizontal and 4 cm in vertical components can be obtained in short baseline mode (~436 m). Montenbruck et al. [9] presented an initial assessment of the positioning performance of BDS. Using BDS observations, they showed that the accuracy of BDS kinematic relative positioning was comparable to that of GPS kinematic positioning, exhibiting a root mean square (RMS) of 3, 3, and 6 mm in the east, north, and vertical components, respectively.

Meanwhile, intensive attention is also paid to PPP with BDS-only observations. Li et al. [10] and Xu et al. [11] realized static and kinematic PPP at centimeter-level accuracy by using BDS observations at a sampling interval of 30 s. Zhao et al. [12] assessed the contributions of BDS GEO, IGSO, and medium Earth orbit (MEO) satellites to PPP in Asia-Pacific region. By using various combinations of different satellite constellations, they showed that BDS IGSO has the largest contribution to the acceleration of convergence and improvement of positioning accuracy to PPP, particularly in the east component. Recently, Li et al. [13] presented that the real-time BDS PPP mode with an accuracy of 5 cm in horizontal and 10 cm in vertical components could be obtained after 3 h convergence.

Moreover, BDS has played an important role in high-precision applications, such as the derivation of the zenith tropospheric delay [14] and the capture of seismic waves. Geng et al. [15] first used 1 Hz BDS observations to measure ground motions induced by the 2015 Mw 7.8 Gorkha, Nepal earthquake. They employed the variometric approach to estimate the velocity time series and to reconstruct displacements by integrating the velocity time series. A novel approach of a linear trend removal was performed to minimize long-period drifts due to mismodeling of different intervening effects during integration to displacements. However, none of the previous studies has been conducted on the monitoring of seismic displacements/waveforms using the BDS PPP approach. With the BDS PPP approach, the seismic displacements are able to be derived directly. As a consequence, the procedure of integration, a requirement of the variometric approach, is successfully circumvented. 

The availability of precise satellite orbit and clock products is of great importance to the realization of PPP. For the GPS constellation, the International GNSS Service (IGS) is able to routinely provide final products containing satellite orbits and clocks with an accuracy of ~2.5 cm and ~75 ps, respectively (see http://igs.org/products). With the advent of new GNSS constellations such as BeiDou, Galileo, Quasi-Zenith Satellite System (QZSS), and Indian Regional Navigation Satellite System (IRNSS), the IGS has initiated the Multi-GNSS Experiment (MGEX) project to pave the way for the provision of high-quality data and products for all GNSS constellations (http://igs.org/mgex/products) [16]. At present, there are three MGEX Analysis Centers (ACs) providing the final satellite orbit and clock products of BDS (i.e., Center for Orbit Determination in Europe (CODE), GeoForschungsZentrum Potsdam (GFZ), and Wuhan University (WHU)) [17]. It is worth noting that CODE only provides the final products of IGSO and MEO. GFZ has been providing 5 min orbit and 30 s clock products instead of 15 min orbit and 5 min clock products since 3 May 2015. 

As shown in Table 1, BDS precise orbit products released by MGEX ACs are updated every 15 min or 5 min, which is similar to GPS precise orbit products. However, the BDS clock products are only provided at 5 min/30 s sampling intervals, while those of GPS are provided at 30 s/5 s intervals. When processing high-rate (up to 1 Hz) GNSS measurements at epochs that do not coincide with the sampling epochs of the clock products, the clock bias of the observed GNSS satellites has to be obtained through interpolation [18,19]. Due to the stochastic characteristic of the clock variations, Montenbruck et al. [20] mentioned that the interpolation error for 5 min clock products would not satisfy the requirement to achieve centimeter-level accuracy for high-rate (up to 1 Hz) GNSS positioning. 

In this contribution, first we generated and evaluated 1 Hz BDS clock corrections based on the precise orbit products. The precision of BDS PPP with 1 Hz clock products was analyzed and applied to retrieve seismic displacements induced by the 2015 Mw 7.8 Gorkha, Nepal, earthquake. Finally, the benefits of high-rate satellite clock corrections to BDS PPP were investigated.

## 2. Data Collection

The 2015 Mw 7.8 Gorkha, Nepal, earthquake took over 9000 lives and injured more than 23,000 people. According to the U.S. Geological Survey (USGS) (http://earthquake.usgs.gov), the origin time of this event was 06:11:26 (UTC), on 25 April 2015; its epicenter was located at 28.147° N, 84.708° E; and its hypocentral depth was 15 km. It was the worst natural disaster to strike Nepal since the 1934 Nepal-Bihar earthquake. Continued aftershocks occurred throughout Nepal at the intervals of 15–20 min. The country also suffered from continuous risk of landslides. The temblor was caused by a sudden thrust, or release of built-up stress, along the major fault line where the Indian plate, carrying India, is slowly diving underneath the Eurasian plate, carrying much of Europe and Asia. Kathmandu, situated on a block of crust approximately 120 km wide and 60 km long, reportedly shifted 3 m to the south in a matter of just 30 s.

The BDS and GPS data were collected and processed to validate the benefit of high-rate satellite clocks for BDS PPP to the measurements of seismic displacements of this earthquake event.

### 2.1. GNSS Stations with 1 s Sampling Rate Data

The station LASA from BDS Experimental Tracking Network (BETN) is located in Lhasa city, 645 km away from the epicenter. The recording of BDS and GPS data at LASA was applied to study the performance of BDS PPP to retrieve high-precision seismic displacements from the Nepal earthquake. 

**Figure 1:**
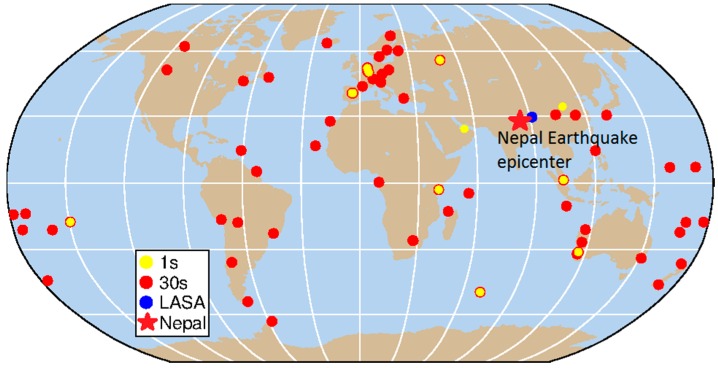
Distribution of selected GNSS stations with 1 s (blue and yellow dots) and 30 s (red dots) sampling intervals data. The star is the epicenter of the 2015 Mw 7.8 Gorkha, Nepal, earthquake.

In order to generate the high-rate GNSS satellite clock products, the data from 1 s GNSS stations from MGEX and BETN were collected and utilized (the blue and yellow dots in Figure 1). BETN network was built and maintained by Wuhan University as a part of continuous global observation network to solve the precise orbit and satellite clock products of BDS in real time. Most stations from BETN are equipped with UB240-CORS receivers manufactured by the Beijing Unicore Communication Company, which can collect both the L1/L2 pseudo-range and phase observation of GPS and B1/B2 of BDS. Two stations from BETN, NTSC in Xi’an and DHAB in United Arab Emirates, can record 1 s observations. Meanwhile, there are 17 stations from MGEX can gather 1 s sampling rate observations of BDS and GPS (ftp://cddis.gsfc.nasa.gov/pub/gps/data/campaign/mgex/highrate/). We chose 10 of these stations that had a good common view of constellation with station LASA, namely: CEBR (Spain), DLF1 (The Netherlands), KRGG (French Southern Territories), KZN2 (Russian Federation), MAL2 (Kenya), NNOR (Australia), REDU (Belgium), SIN1 (Singapore), THTG (French Polynesia), VILL (Spain).

### 2.2. GNSS Stations with 30 s Sampling Rate Data

In April 2015, four stations from BETN were recording 30 s sampling rate data: CHDU in Chengdu, LEID in The Netherlands, PFTP in Australia, and JOHA in South Africa. Meanwhile, MGEX has about 60 stations which can record BDS and GPS observations at 30 s intervals. In addition to selected 13 stations sampling at a 1 s rate and 68 stations sampling at a 30 s rate (shown in Figure 1) were added as reference tracking data to implement the precise orbit determination (POD) procedure to obtain higher precision satellite orbits.

## 3. BDS Precise Orbit and Clock Products Generation

There are currently 30 s BDS clocks provided by GFZ (see Table 1), but they were not yet available at the time of the earthquake. Since the precision of low sampling rate (5 min) of clock corrections can hardly meet the requirement of high-rate high-precision positioning, BDS precise orbits and high-rate clock products were produced in this study. In this section, we first present the process strategy of BDS POD and PCE (precise clock estimation), and then evaluate the orbit and clock products.

### 3.1. Processing Strategy

The Position and Navigation Data Analyst (PANDA) developed by GNSS Research Center of Wuhan University [21] was employed to conduct POD, PCE, and PPP in this study. Table 2 summarizes the observation models, dynamical models, and estimated parameters for POD and PCE.

BDS orbital parameters were generated only from BDS observations and fully independent from the other GNSS observations. Besides, epoch-wise satellite and receiver clock offsets, station coordinates, zenith troposphere delay (ZTD), as well as float ambiguities for each satellite were also estimated. There are 68 GNSS stations with 30 s intervals in Figure 1, and their observations from 22 to 28 April were processed. We took three consecutive days as one orbit arc in least-squares estimation. Zero-difference ionosphere-free observations of B1 and B2 phase and code observations were used to eliminate the ionosphere delays effect. The data processing interval was 30 s and the elevation cutoff angle was 7°. To detect bad measurements and remaining cycle slips, raw measurements were further cleaned by analyzing posterior residuals derived from the estimation. The prior orbits were originated from the broadcast ephemeris as initial values. NNOR with an external atomic clock was selected as the reference clock. The BDS orbit and 30 s clock corrections were obtained synchronously, which were called BDS WHU orbits and WHU 30 s clocks, respectively.

For the procedure of PCE, the BDS orbits and station coordinates were held fixed. GPS orbits came from the IGS final products. Besides satellite clocks, station clocks, ZTD, as well as float ambiguities were estimated in the PCE procedure. The BDS and GPS 1 s satellites’ clock products were generated by the 1 s GNSS data from 13 stations, shown in Figure 1. Complete 24 h GNSS data starting from 10:00:00 24 April 2015 were processed. Initial satellite clocks were obtained from the broadcast ephemeris. It should be mentioned that the satellites clock corrections of BDS were processed independently, and the corresponding solutions were called WHU 1 s clock.

### 3.2. Quality of the BDS WHU Orbits 

In order to externally evaluate BDS WHU orbits, two different methods presented in Steigenberger et al. [26] were employed: (1) comparison with the other ACs and (2) satellite laser ranging (SLR) validation.

#### 3.2.1. Comparison with the Other ACs

The most straightforward indicator for assessing orbit quality is the consistency between different ACs. BDS orbits are compared at 15 min intervals in the along-track, cross-track, and radial directions. Since the length of POD arc was 3 days, only the orbital solutions of the middle day were used for validation. The BDS WHU orbits were compared with other MGEX ACs (i.e., GBM and COM (only BDS IGSO and MEO)).

**Figure 2:**
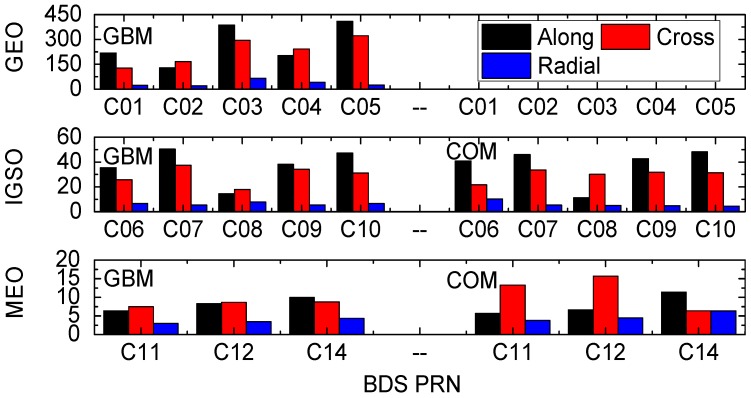
RMS of differences of BDS WHU orbit compared with GBM and COM in along-track, cross-track, and radial directions (unit: cm). PRN: Pseudo Random Noise.

It is clear from Figure 2 that the GEO orbits show the worst quality, while the MEO orbits show the best quality. The RMS values of orbit differences among GBM, COM, and WHU are shown in Table 3. For GEOs, RMS of orbit differences between WHU and GBM are up to nearly 0.3 m in the radial direction, whereas the values in the cross-track and along-track directions are about 2 m and 3 m, respectively. For IGSOs, the orbits of GBM, COM, and WHU agree well, with RMS values of the orbit differences less than 0.4 m, 0.3 m, and 0.1 m in along-track, cross-track, and radial directions, respectively. For MEOs, the orbits by GBM, COM, and WHU show the best agreement, with an RMS of about 0.05–0.10 m in the along-track, 0.06–0.15 m in cross-track, and 0.03–0.06 m in the radial component. 

#### 3.2.2. SLR Residuals

The BDS C01 (GEO), C08 (IGSO), C10 (IGSO), and C11 (MEO) satellites are equipped with laser retroreflector arrays, which enabled us to independently validate the orbit accuracy with SLR residuals. The SLR residuals (i.e., the differences between observed SLR values and the computed distance using the GNSS orbits and references stations) were computed during the experiment period. Only the orbital solutions of the middle day were used for validation. 

Table 4 shows the number of normal points and statistic values of biases, standard deviation (STD) and RMS, of SLR residuals of the BDS WHU orbit solutions. The RMS of the residuals is 75.7 cm for C01, 7.5 cm for C08, 4.0 cm for C10, and 2.6 cm for C11. It is worth noting that the SLR residuals are orbit errors projected into the line-of-sight of SLR observations. Thus, they include three components of orbit errors, not only the radial component. The SLR residuals of C01 are much larger than the other three satellites. It is well known that GEO satellites have larger orbit errors, especially in the along-track and cross-track directions.

#### 3.2.3. Quality of BDS WHU 1 s Clocks 

In order to evaluate the precision of BDS WHU 1 s clocks, the double-difference approach has been employed. In the first step, the differences of other satellite clocks with the reference clock (BDS C06) from the same ACs have been calculated to remove the clock systematic biases. We choose C06 as a reference because it could be tracked with a relatively long visible arc, considering the coverage of the 1 s regional tracking network (13 stations marked by yellow dots in Figure 1). Actually, the clock comparison results are independent of the choice of the reference satellite clock if the satellite clock is stable. Secondly, the clock differences of the same satellite pairs from different ACs were measured. It is noted that only the epochs of satellites’ elevations with respect to LASA station higher than 7° are used. The corresponding STD of the double-difference clocks were used as indicators of clock quality. The STD of clock differences have been calculated for all available satellites using the following formula:

$$\mathrm{STD}=\sqrt{\frac{\sum_{i=1}^{n}\left(\Delta_{i}-\bar{\Delta }\right)\left(\Delta_{i}-\bar{\Delta }\right)}{n}}$$

where $\Delta_{i}$ is the doule-difference of the WHU 1 s clock with the others clock (i.e., WHU 30 s, GBM, COM) at epoch i, $\bar{\Delta }$ is the average bias, and n refers to the number of epochs. 

To assess the precision of WHU 1 s clocks, we conducted external and internal evaluations, respectively. For the external evaluation, we compared WHU products with COM and GBM. Since they only have 300 s clock products (see Table 1), we resampled our 1 s clocks to 300 s intervals before comparison. 

**Figure 3:**
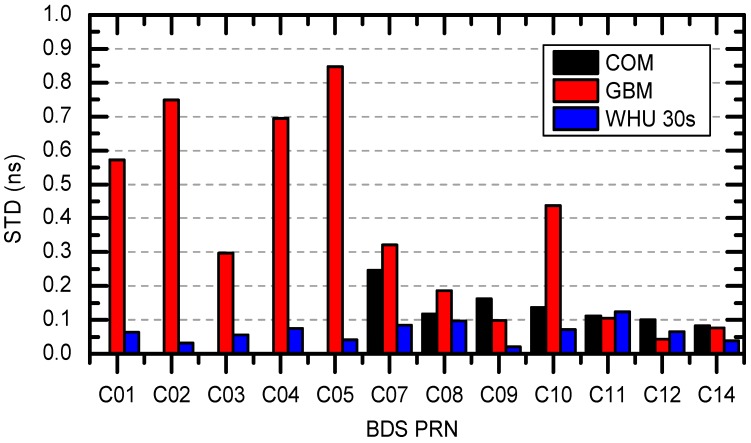
Comparisons of BDS WHU 1 s clocks with respect to COM, GBM, and WHU 30 s products.

Figure 3 shows the STD values of satellite-specific clock differences of BDS WHU 1 s products with respect to COM and GBM products. The clock errors were also calculated in terms of three satellite types—GEO, IGSO, and MEO—and listed in Table 5. The clock errors of GEO are relatively large compared with the errors of IGSO and MEO. The RMS values of GEO clock errors could reach up to 0.63 ns, whereas the RMS of IGSO and MEO clock errors are about 0.2–0.3 ns and 0.1 ns, respectively.

For the internal evaluation, the WHU 1 s clock was compared at 30 s intervals with WHU 30 s clock, similarly. As is shown in last column of Table 5, WHU 1 s and WHU 30 s products are almost the same, with STD of differences less than 0.1 ns. The comparison not only proves that WHU 1 s clocks show a high degree of consistency with WHU 30 s clocks, but also indicates that 13 regional stations could achieve the same accuracy as 68 global stations in the China-Australia area.

## 4. Application to Earthquake Monitoring

### 4.1. PPP Prior to the Earthquake

In order to assess the accuracy of GNSS PPP, 1 Hz BDS and GPS data at station LASA over a 12 h time span (from 10:00 to 22:00) on 24 April 2015 (prior to the earthquake event, and the station is stationary) was processed. Dual-frequency, ionosphere-free, pseudo-range, and carrier phase combinations with an elevation angle cutoff of 7° were used. BDS satellite orbits and clocks were held fixed to the WHU orbit and WHU 1 s clock, while the IGS final orbit and WHU 1 s clock were fixed for GPS. Epoch-wise station coordinates and clock, tropospheric zenith path delay, as well as float ambiguities are estimated. The observation models, dynamical models, and estimated parameters are also given in Table 2.

**Figure 4:**
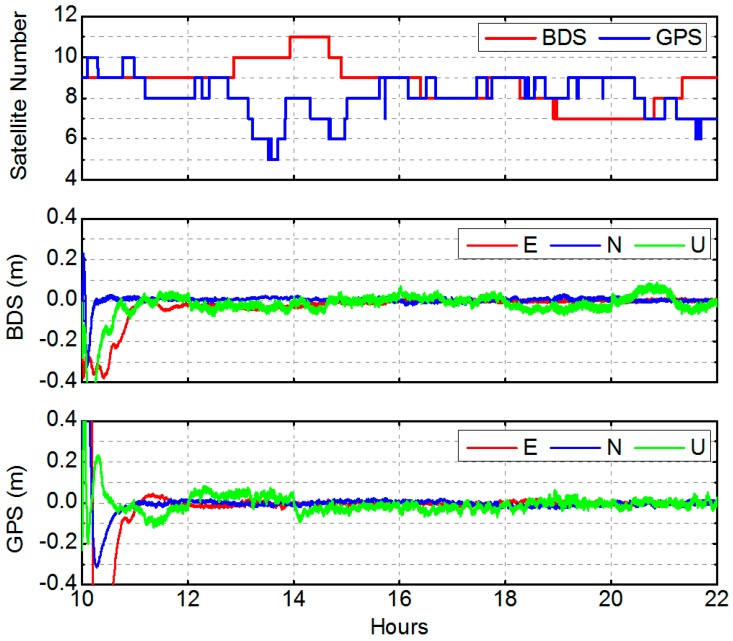
Number of observed satellites and the times series of PPP results with BDS and GPS (LASA station, from 10:00:00 UTC, 24 April 2015).

The visible number of satellites and results from BDS PPP and GPS PPP solutions are shown in Figure 4. Average numbers of observed satellites of GPS and BDS are almost the same, which is 8.1 and 8.4, respectively. RMS of BDS PPP and GPS PPP solution errors over the period from 14:00 to 22:00 (after the convergence) have been calculated (Table 6). For BDS solutions, the horizontal RMS is better than 1 cm and consistent with GPS, while the vertical reaches up to 5 cm, somewhat worse than GPS. The somewhat worse vertical performance of BDS PPP is mostly due to the slightly worse orbit and clock precision of the BDS GEO satellites.

### 4.2. PPP during the Earthquake

**Figure 5:**
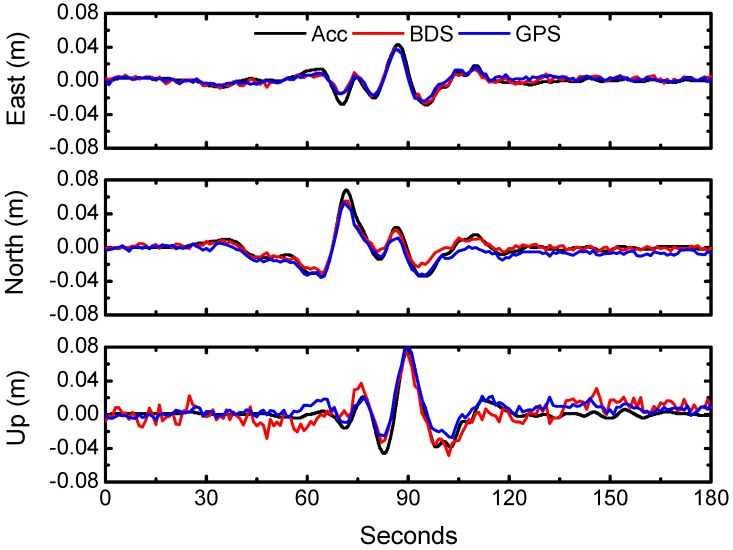
The displacement waveforms from GPS PPP and BDS PPP at LASA and from strong motion station LSA over the period from 6:14:00 to 6:17:00 (UTC).

The seismic displacements associated with the earthquake at station LASA were retrieved using BDS PPP and GPS PPP, respectively. As shown in Figure 5, the displacements from BDS PPP are consistent with those from GPS PPP; the RMS of differences between GPS and BDS displacements are 0.29, 0.38, and 1.08 cm in east, north, and vertical components, respectively. It is worth noting that the RMS of differences during the earthquake is calculated from only 3 min length of data (from 6:14:00 to 6:17:00 UTC). As a comparison, we randomly took five segments of data, each with a length of 3 min, to demonstrate the differences between GPS and BDS solutions for the period before the earthquake. We also computed the RMS for all three components, which are listed in Table 7. The average RMS values in Table 7 clearly show that the differences between GPS and BDS solutions before the earthquake are highly consistent with those from the data during the earthquake.

Meanwhile, there is a strong motion station, LSA, which records accelerometer data, located closely to station LASA, with a distance of 5.5 km. To further validate the reliability of the displacements derived from LASA, we also derived the displacements from accelerometer recordings observed at station LSA with a double integration and baseline correction scheme described in [27]. Figure 5 shows good agreement between the GNSS and strong motion instruments during the fluctuation associated with the earthquake.

## 5. BDS PPP with Different Clock Interval

Due to the stochastic characteristic of the clock variations, the interpolation of the low-rate satellite clock products has a relatively large loss of precision. As a result, it is not sufficient for high-rate (up to 1 Hz) precise positioning and applications. When processing 1 Hz measurements at epochs that do not coincide with sampling epochs of the ephemeris products, the orbit and clock offset of the observed satellites have to be obtained through interpolation. In contrast to orbit data, high-order polynomial interpolation is not suitable for clock parameters due to the underlying random noise processes; therefore, linear interpolation is advisable [20].

We utilized BDS WHU 1 s clock solutions to assess the errors associated with the interpolation of 5 s, 30 s, and 300 s clock data sets. Firstly, we resampled the BDS WHU 1 s clock products to 5 s, 30 s, and 300 s interval products. Secondly, the 5 s, 30 s, and 300 s data sets were interpolated to a 1 s interval, respectively. Finally, they were compared with the 1 Hz products using the double-difference approach described in Section 3.

The 1 s WHU clocks were estimated based on the 13 GNSS stations with 1 s intervals, which are mainly deployed in the China-Australia area. When we calculated the interpolation errors, we took station LASA as a reference. In other words, we only compared the tracking arcs (epochs) that are visible in LASA station with elevation cutoff angle of 7°. 

**Figure 6:**
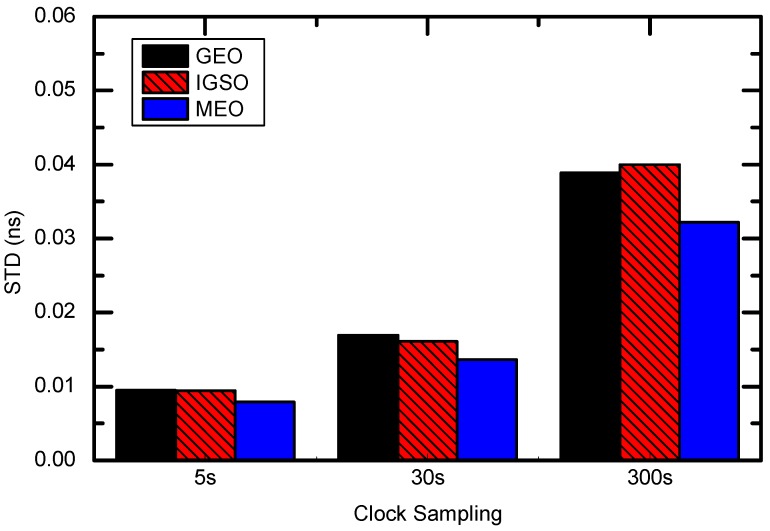
STD of BDS clock interpolation error for 5 s, 30 s, and 300 s intervals.

The clock interpolation errors are summarized in Figure 6 for the individual clock types. The best interpolation results are obtained from 5 s intervals. The largest errors are obtained from 300 s intervals. For 30 s intervals, the interpolation errors are better than 0.02 ns (i.e., 0.6 cm), while for 300 s intervals, the interpolation errors increase to 0.04 ns (1.2 cm) level.

**Figure 7:**
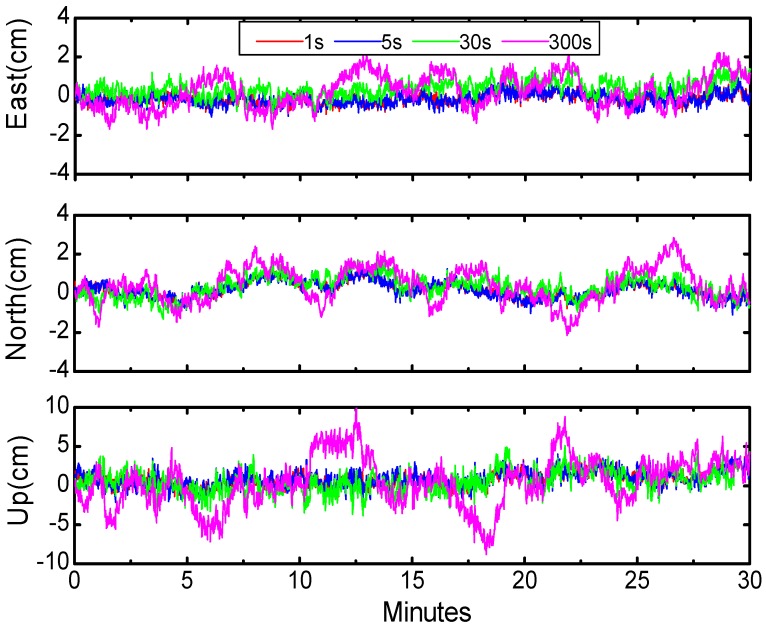
Accuracy of BDS PPP in the east, north, and vertical components with clock intervals of 1 s, 5 s, 30 s, and 300 s, starting from 15:00:00 (UTC) 24 April 2015.

To further investigate the contribution of clock corrections with different sampling intervals, we conducted BDS PPP to process the same data as Section 4 by applying satellite clock products at 300 s, 30 s, 5 s, and 1 s intervals, respectively. To demonstrate the accuracy of BDS PPP solutions (prior to the earthquake), we randomly took five segments of BDS data, each with a length of 30 min, and computed the RMS of BDS PPP with clock intervals of 1 s, 5 s, 30 s, and 300 s for all four cases, which are listed in Table 8. Shown in Figure 7 is one segment of the error performances of BDS PPP in the east, north, and vertical components with clock intervals of 1 s, 5 s, 30 s, and 300 s respectively. From the mean values of Table 8, it could be concluded that the PPP with 300 s clock intervals is the worst and that with 1 s clock interval is the best. Comparing the results with 1 s and 5 s clock intervals, they have almost the same precision, which means that the contribution of 5 s clock intervals are almost equal to 1 s clock interval.

**Figure 8:**
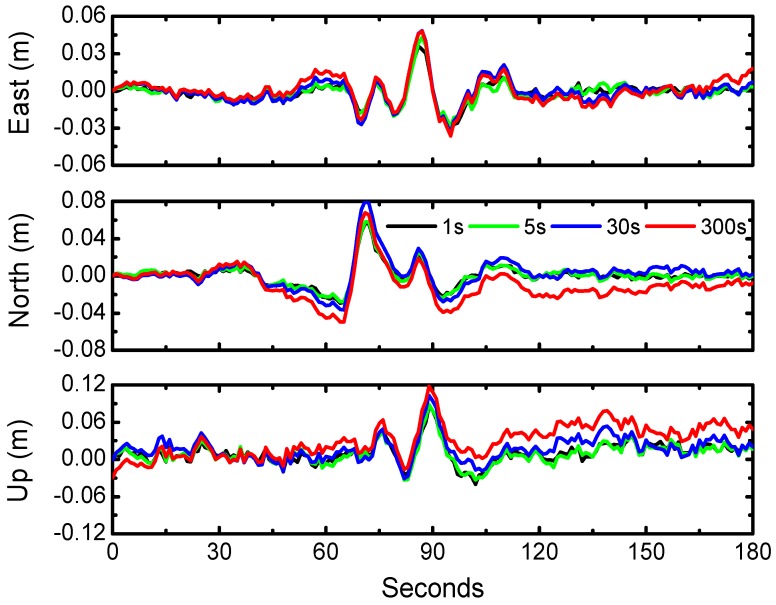
The displacement waveforms from BDS PPP with 1 s, 5 s, 30 s and 300 s clock intervals over the period from 6:14:00 to 6:17:00 (UTC).

In addition, we retrieved the displacements waveforms with BDS PPP (during the earthquake) using different clock products at intervals of 1 s, 5 s, 30 s, and 300 s (Figure 8); results clearly show that 1 s and 5 s clock product demonstrate more stable and reliable waveforms. Considering the time consumption of clock estimates, we suggest that 5 s clock interval is competent for the requirement of high-rate BDS solutions. This phenomenon is consistent with that derived from GPS PPP results [18].

## 6. Conclusions 

BDS PPP have been implemented and employed to retrieve seismic displacements of the 2015 Mw 7.8 Gorkha, Nepal, earthquake. In order to obtain positioning results with higher precision, 1 Hz BDS satellite clock corrections were generated based on precise orbit products from WHU. They were utilized for BDS PPP solutions and their results were compared with the counterpart of GPS PPP. It is demonstrated that the accuracy of BDS PPP could reach 1 cm in horizontal and 2 cm in vertical components in retrieving seismic displacements of this earthquake event, which is comparable to the result of GPS PPP. 

We have also analyzed the contributions to BDS PPP of clock corrections with different intervals. It is shown that PPP with 300 s clock intervals is the worst and that with 1 s clock interval is the best, while the precision of PPP solutions for the 5 s clock intervals is almost the same as 1 s results. Considering the time consumption of clock estimates, we suggest that the 5 s clock interval is adequate with regard to the requirement of high-rate BDS solutions. 

It should be noted that the approach of clock generation in this study is not suitable for real-time estimation. For future research we plan to investigate the real-time clock estimation approach for real-time applications, such as rapid determination of earthquake magnitude [28], earthquake early warning [29], and rapid hazard assessment [30].

## Figures and Tables

**Table 1:** An overview of the MGEX BDS precise orbit and clock products.

Institution	ID	Orbit	Clock	Remarks
CODE	COM	15 min	5 min	Since October 2013
GFZ	GBM	5 min	30 s	Since 3 May 2015
WHU	WUM	15 min	5 min	Since January 2013

MGEX: Multi-GNSS Experiment; BDS: BeiDou Navigation Satellite System CODE: Center for Orbit Determination in Europe; COM: the GNSS products from CODE; GFZ: GeoForschungsZentrum Potsdam; GBM: the GNSS products from GFZ; WHU: Wuhan University; WUM: the GNSS products from WHU.

**Table 2:** Observation models, dynamical models, and estimated parameters for precise orbit determination (POD), precise clock estimation (PCE), and precise point positioning (PPP) in Position and Navigation Data Analyst (PANDA) software.

Item	Models
Observations Combination	Ionosphere-free code and carrier phase combination
Carrier phase signal	Global Positioning System (GPS): L1/L2, BDS: B1/B2
Sampling rate	30 s for POD, 1 s for PCE and PPP
Elevation cutoff	7°
Observation weight	0.002 m and 2.0 m for raw phase and code observables, respectively, and elevation-dependent data weighting
Satellite antenna phase center	Corrected using MGEX values [22]
Receiver antenna phase center	Corrected using GPS values [22]
Phase-windup effect	Phase polarization effects applied [23]
Troposphere delay	Saastamoinen model for wet and dry hydrostatic delay with Global Mapping Function (GMF), estimated as piecewise constant function with 2 h parameter for residual wet delay
Ionosphere delay	First-order effect eliminated by forming the ionosphere-free combinations
Station displacement	Solid Earth tide, pole tide, ocean tide loading, International Earth Rotation and Reference Systems Service (IERS) Convention 2003 [24]
Satellite orbit	Estimated in POD; fixed in PCE and PPP
Satellite clock	Estimated in POD and PCE, white noise; fixed in PPP
Receiver clock	Estimated as random walk process, NNOR as reference clock
Station coordinate	Tightly constrained for POD; fixed for PCE; estimated in epoch-wise kinematic mode for PPP
Phase ambiguities	Real constant value for each ambiguity arc
EOP parameters	Polar motions and UT1 from IERS C04 series aligned to International Terrestrial Reference Frame (ITRF) 2008
Attitude model	Nominal attitude with yaw maneuver for medium Earth orbit (MEO) and inclined geosynchronous orbit (IGSO), yaw-fixed attitude mode used for geostationary Earth orbit (GEO) [13]
Geopotential	EIGEN_GL04C up to 12 × 12
Tide	Solid Earth tide, pole tide, ocean tide, IERS Conventions 2003
N-body gravitation	Sun, Moon, and other planets; JPL DE405 ephemeris used
Relativity effect	IERS Conventions 2003
Solar radiation	ECOM(Extended CODE Orbit Model) model 5-parameter with no initial value [25]

**Table 3:** Root mean square (RMS) of BDS orbit comparisons among GBM, COM, and WHU (unit: cm).

	GBM vs. WHU			COM vs. WHU			COM vs. GBM		
	Along	Cross	Radial	Along	Cross	Radial	Along	Cross	Radial
**GEO**	268.9	230.5	34.5	-	-	-	-	-	-
**IGSO**	37.1	29.3	6.3	37.8	29.7	6.0	9.6	16.7	7.8
**MEO**	4.9	5.4	2.1	4.1	9.6	2.7	9.4	11.7	6.7

**Table 4:** Numbers of normal points (NPs) and statistic values of biases, standard deviation (STD) and RMS, of satellite laser ranging (SLR) residuals of BDS WHU orbits (unit: cm).

PRN	NPs	WHU			COM			GBM		
		AVE	STD	RMS	AVE	STD	RMS	AVE	STD	RMS
C01	9	−73.9	16.3	75.7				−102.0	12.8	102.8
C08	17	−2.7	7.0	7.5	0.2	8.9	8.9	12.7	9.4	15.8
C10	22	−0.9	3.9	4.0	−6.7	6.8	9.6	−11.3	8.1	13.9
C11	35	−0.7	2.5	2.6	−3.9	8.2	9.1	−0.9	8.1	8.1

**Table 5:** Statistic RMS values of BDS WHU 1 s clocks with respect to COM, GBM, and WHU 30 s clocks in term of three satellite types of GEO, IGSO, and MEO (unit: ns).

Types	WHU 1 s vs. COM	WHU 1 s vs. GBM	WHU 1 s vs. WHU 30 s
GEO		0.63	0.05
IGSO	0.17	0.26	0.07
MEO	0.10	0.08	0.08

**Table 6:** RMS of BDS PPP and GPS PPP solution errors over the period from 14:00 to 22:00 (unit: cm).

Mode	East	North	Up
BDS-only	0.6	0.8	4.3
GPS-only	0.9	0.9	2.8

**Table 7:** RMS of differences between GPS and BDS solutions for the period before the earthquake with 3 min length of data (unit: cm).

Sessions (3 min)	East	North	Up
1	0.47	0.35	1.25
2	0.32	0.29	1.10
3	0.39	0.35	0.81
4	0.40	0.39	0.94
5	0.42	0.37	1.14
Ave	0.40	0.35	1.05

**Table 8:** RMS of BDS PPP with clock intervals of 1 s, 5 s, 30 s, and 300 s (unit: cm).

Sessions	1	2	3	4	5	Mean
East component						
1 s	0.28	0.63	0.29	0.30	0.32	0.36
5 s	0.32	0.64	0.32	0.32	0.35	0.39
30 s	0.43	0.75	0.58	0.56	0.43	0.55
300 s	0.83	1.02	0.70	0.72	0.92	0.84
North component						
1 s	0.41	0.64	0.68	0.57	0.62	0.58
5 s	0.43	0.65	0.72	0.63	0.65	0.62
30 s	0.55	0.69	0.92	0.96	0.77	0.78
300 s	0.90	0.98	1.38	1.58	1.09	1.19
Up component						
1 s	0.96	1.17	1.31	1.41	1.11	1.19
5 s	1.12	1.24	1.35	1.46	1.18	1.27
30 s	1.46	1.50	1.86	1.49	1.68	1.60
300 s	3.15	2.95	3.01	2.85	2.15	2.82
